# Simulating PM_2.5_ Concentrations during New Year in Cuenca, Ecuador: Effects of Advancing the Time of Burning Activities

**DOI:** 10.3390/toxics10050264

**Published:** 2022-05-19

**Authors:** René Parra, Claudia Saud, Claudia Espinoza

**Affiliations:** 1 Instituto de Simulación Computacional (ISC-USFQ), Colegio de Ciencias e Ingenierías, Universidad San Francisco de Quito USFQ, Quito 170901, Ecuador; csaud@usfq.edu.ec; 2 Red de Monitoreo de Calidad del Aire de Cuenca, Empresa Pública de Movilidad, Tránsito y Transporte de Cuenca, EMOV EP, Cuenca 010206, Ecuador; cespinoza@emov.gob.ec

**Keywords:** WRF-Chem, air quality modeling, fine particles, planetary boundary layer, atmospheric stability

## Abstract

Fine particulate matter (PM_2.5_) is dangerous to human health. At midnight on 31 December, in Ecuadorian cities, people burn puppets and fireworks, emitting high amounts of PM_2.5_. On 1 January 2022, concentrations between 27.3 and 40.6 µg m^−3^ (maximum mean over 24 h) were measured in Cuenca, an Andean city located in southern Ecuador; these are higher than 15 µg m^−3^, the current World Health Organization guideline. We estimated the corresponding PM_2.5_ emissions and used them as an input to the Weather Research and Forecasting with Chemistry (WRF-Chem 3.2) model to simulate the change in PM_2.5_ concentrations, assuming these emissions started at 18:00 LT or 21:00 LT on 31 December 2021. On average, PM_2.5_ concentrations decreased by 51.4% and 33.2%. Similar modeling exercises were completed for 2016 to 2021, providing mean decreases between 21.4% and 61.0% if emissions started at 18:00 LT. Lower mean reductions, between 2.3% and 40.7%, or even local increases, were computed for emissions beginning at 21:00 LT. Reductions occurred through better atmospheric conditions to disperse PM_2.5_ compared to midnight. Advancing the burning time can help reduce the health effects of PM_2.5_ emissions on 31 December.

## 1. Introduction

Fireworks to welcome in the new year are customary across different parts of the world [1,2]. Around midnight on 31 December in Ecuador, it is also a tradition to burn the “años viejos”, cloth dolls filled with sawdust or paper. Both the fireworks and the burning of puppets take place in a festive environment amid social and family connections [3]. The use of fireworks results in raising short-term particulate matter concentrations [4], which affects public health and visibility [5], reaching levels that typically exceed both national regulations and the guidelines of the World Health Organization (WHO). Most of the particles aerosolized by burning fireworks consist of metals and compounds in colorful firework displays [2].

PM_2.5_ produces health effects after both short-term and long-term exposure. The WHO guideline does not guarantee complete protection against PM_2.5_ effects [6], requiring the lowest concentrations to be achieved. Short-term exposure promotes cardiovascular and respiratory effects [7]. The International Agency for Research on Cancer (IARC) classified particulate matter and outdoor air pollution as carcinogenic to humans [8,9]. Moreover, in terms of effects on the brain, particulate matter appears to be the most concerning air pollutant [10]. PM_2.5_ accumulates in the reproductive organs, disrupting hormone levels and affecting fertility [11]. Moreover, pyrotechnic fireworks introduce significant quantities of toxic metals into the atmosphere, affecting mammalian cells and lungs [12]. PM_2.5_ affects red blood cell counts, monocyte counts, and hemoglobin concentration [13]. Fireworks have increased health problems, particularly in infants, women (including pregnant women), the elderly, and fetuses. Children suffer from an increased risk of diseases due to exposure to heavy metals emitted by pyrotechnics [14]. Short-term exposure to PM_2.5_ was recently associated with an increased risk of and mortality associated with myocardial infarction in patients with acute coronary syndromes and angina [15]. Comparatively, exposure to PM_1_ exhibited a higher risk for emergent department visits than PM_2.5_ [16], and PM_1_ may be more hazardous to children’s respiratory systems than PM_2.5_ exposure [17]. Although air pollution from New Year’s emissions occurs once a year, it can be a potential risk, especially for people with pre-existing diseases [18]. Therefore, particulate matter levels in the atmosphere, both at short- and long-term concentrations, need to be controlled worldwide.

Particulate matter (PM) concentrations in ambient air are the result of a complex two-way interaction between emissions and atmospheric physical–chemical processes [19,20]. The dynamic of the planetary boundary layer (PBL)—the turbulent bottom layer of the atmosphere, which experiences a daily cycle of depth, temperature, wind, and pollution variations—directly influences air pollutant concentrations. If the atmosphere shows unstable conditions, as at sunny middays, PBL depth will be high, promoting PM dispersion [21]. On the other hand, if the atmosphere is stable, as it is during strong thermal inversion, PBL depth will be shallow, and particulate matter will remain concentrated. Radiation, cloud cover, wind speed, and direction also influence the behavior of air quality [22,23]. Other processes, such as accumulation, condensation, fragmentation, and sedimentation, also influence PM concentrations [19,24,25,26].

Emissions from fireworks, firecrackers, and puppet burning are typically highest during the first hours of a new year. In Cuenca (Ecuador), typically the PM_2.5_ concentrations at the beginning of the year are higher than the WHO’s guidelines (e.g., [27,28,29]), implying the effects on public health reported in the literature [5,7,12,14,15], and therefore, the need to reduce PM_2.5_ concentrations. Recently, the air quality network from Cuenca began to measure both PM_2.5_ and PM_1_, which is valuable information for improving understanding of the air quality problems in this city and how atmospheric pollution is affecting public health.

Although the direct way to control PM_2.5_ levels is to reduce emissions by limiting combustion sources, implementing firework prohibition policies during the Chinese Lunar New Year’s celebrations did not result in clear air quality benefits [30]. Another option is to manage the corresponding emission factors (the amount of PM_2.5_ emitted per unit of pollutant activity), promoting the use of environmentally friendly fireworks. However, this alternative must be accompanied by strict control of the number of used fireworks [31]. Due to these limitations, it is necessary to study other options to reduce air pollution by New Year’s emissions. We did not find dedicated studies for this purpose in Ecuador, and therefore, exploring alternatives to reduce PM_2.5_ levels at the beginning of the year is necessary.

At midnight, the temperature and wind speeds in Cuenca usually present lower values compared with the preceding hours. Low temperature and wind speed values are associated with low PBL depths [21] and stronger atmospheric stability [32]. Although weather conditions cannot be controlled, another novel option could be to advance the burning of fireworks and puppets to a time when, compared with midnight, the atmosphere presents better conditions to disperse air pollutants. Hence, this study reports the PM_1_ and PM_2.5_ records on 31 December 2021 and 1 January 2022. Moreover, we performed numerical experiments to estimate the changes in PM_2.5_ concentrations to answer the following questions:

- Are there reductions in PM_2.5_ concentrations if the emission activities associated with the arrival of a new year in Cuenca occur some hours before midnight?
- What is the magnitude of these changes?

## 2. Materials and Methods

### 2.1. Primary Sources and Past PM_2.5_ Concentrations in Cuenca

**Figure 1:**
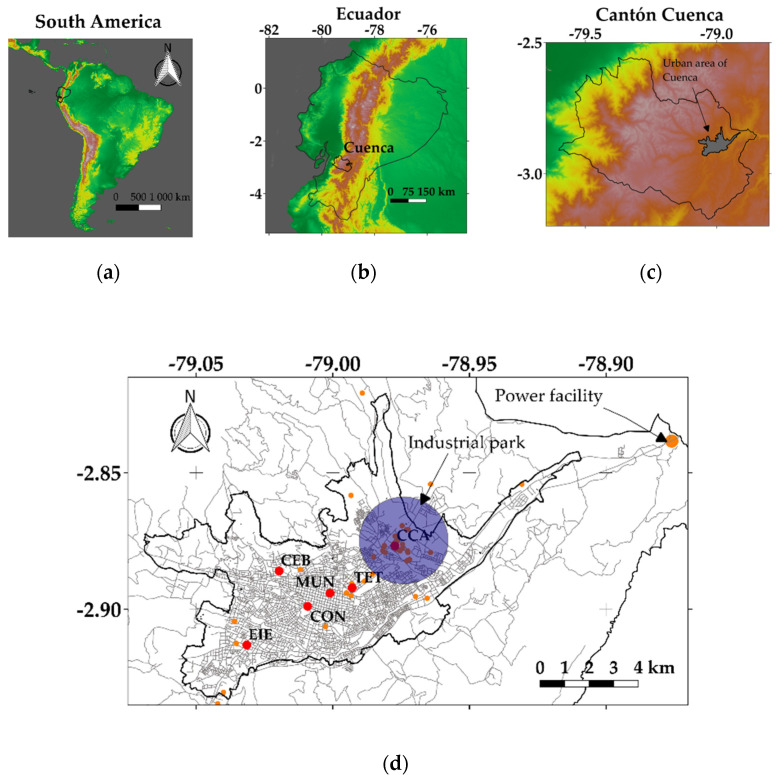
Location of: (**a**) Ecuador; (**b**,**c**) Cuenca; (**d**) urban area of Cuenca and the air quality stations (red dots) with PM_2.5_ sensors (name, nomenclature): Colegio Carlos Arízaga, CCA; Terminal Terrestre, TET; Municipio, MUN; Condamine, CON, Cebollar, CEB; Escuela Ignacio Escandón, EIE. Orange dots indicate industries.

Cuenca (2500 m.a.s.l.), an Andean city located in southern Ecuador (Figure 1), has a complex topography, with altitudes ranging from 1000 to 4000 m.a.s.l. This city has approximately 640,000 inhabitants, representing approximately 3.6% of the Ecuadorian population [33]. This territory has diverse land-use categories. The primary sources of PM_2.5_ are diesel vehicles, industries at the northeast zone of the city, and a power facility located in the northeast of the urban area (Figure 1d). Moreover, irregular PM_2.5_ emissions come from artisanal brick production in the northwest of the urban region, due to the combustion of biomass [34]. More information about the magnitude of the emission sources in Cuenca are described in Parra (2018) [35] and Parra and Espinoza (2020) [36].

During the first hours of 2016 to 2021, the air quality network in the MUN (Municipio) station measured maximum hourly PM_2.5_ concentrations between 55.8 and 182.1 µg m^−3^. The MUN station has been operating since 2012, measuring both meteorological parameters and air quality. Some years later, the Colegio Carlos Arízaga (CCA) and Escuela Ignacio Escandón (EIE) stations were installed to monitor air quality in the zones of the industrial park and in the southwest of the city (Figure 1d). In the last weeks of 2021, three new stations were installed (Condamine, CON; Terminal Terrestre, TER; Cebollar, CEB), equipped with sensors to record PM_2.5_ and PM_1_. The air quality network is operated by the Municipality of Cuenca, the entity accredited by the National Environmental Authority.

### 2.2. Estimation of PM_2.5_ Emissions through Burning Activities Associated with the Arrival of 2022

In general, emission inventories have high levels of uncertainty. This feature is especially critical when estimating New Year’s emissions. After exploring different approaches, we estimated the total PM_2.5_ emissions at the beginning of 1 January 2022 as the contribution of the use of fireworks and the burning of puppets, through the basic emission model:

(1) $${\mathrm{PM}}_{2.5}\;\mathrm{Emission}=\mathrm{Activity}\;\times \;\mathrm{Emission}\;\mathrm{Factor}$$

Activity: amount of fireworks or puppets burned during the New Year’s festivities. Emission factor: amount of PM_2.5_ emitted by each firework or puppet burned.

#### 2.2.1. Emissions Due to the Use of Fireworks

Firstly, we estimated the amount of fireworks used in Cuenca, through the following approach.

Based on information about the amount of money spent for the importation of fireworks in 2021 [37,38], we estimated that in Cuenca, approximately USD 112,000 was allocated for the purchase of fireworks in December. We identified the pyrotechnic cake with 30 shots, which has approximately 500 g of net explosive content (NEC), as the most consumed product. Using an average price of USD 24 per cake, we estimated that there was 3497 kg NEC in the amount of fireworks legally sold. Moreover, we considered the additional amount of fireworks from illegal sales by non-controlled producers or importers. Based on information about illegal fireworks [39] and an assumed percentage of non-controlled sales, we estimated a 3279 kg NEC as a result of these sales. Therefore, the total amount of fireworks were estimated to equal 6776 kg NEC.

Then, we selected the average emission factor of 200 g PM_2.5_ NEC^−1^ proposed by Keller and Schragen (2021) [40] for common pyrotechnic articles. Applying Equation (1), the corresponding emission was 1.36 t PM_2.5_.

#### 2.2.2. Emissions Due to the Burning of Puppets

The amount of puppets burned was estimated through the following approach.

The Municipality of Cuenca determined 369 authorized points for selling puppets, with an average amount of 25 units sold per day [41]. Considering the number of sales over two days, an average weight of 2 kg per unit, and an increase of 25% due to domestic production and non-authorized sales, we estimated 46.1 t as the amount of biomass contained in the puppets. Although in recent years authorities have disincentivized the use of sawdust, it is still used for filling puppets. Therefore, we selected the average emission factor of 8.10 g PM_2.5_ per kg of biomass, proposed for wood waste in the European emission factor database [42]. Therefore, the estimated emission due to the burning of puppets was 0.37 t PM_2.5_.

**Figure 2:**
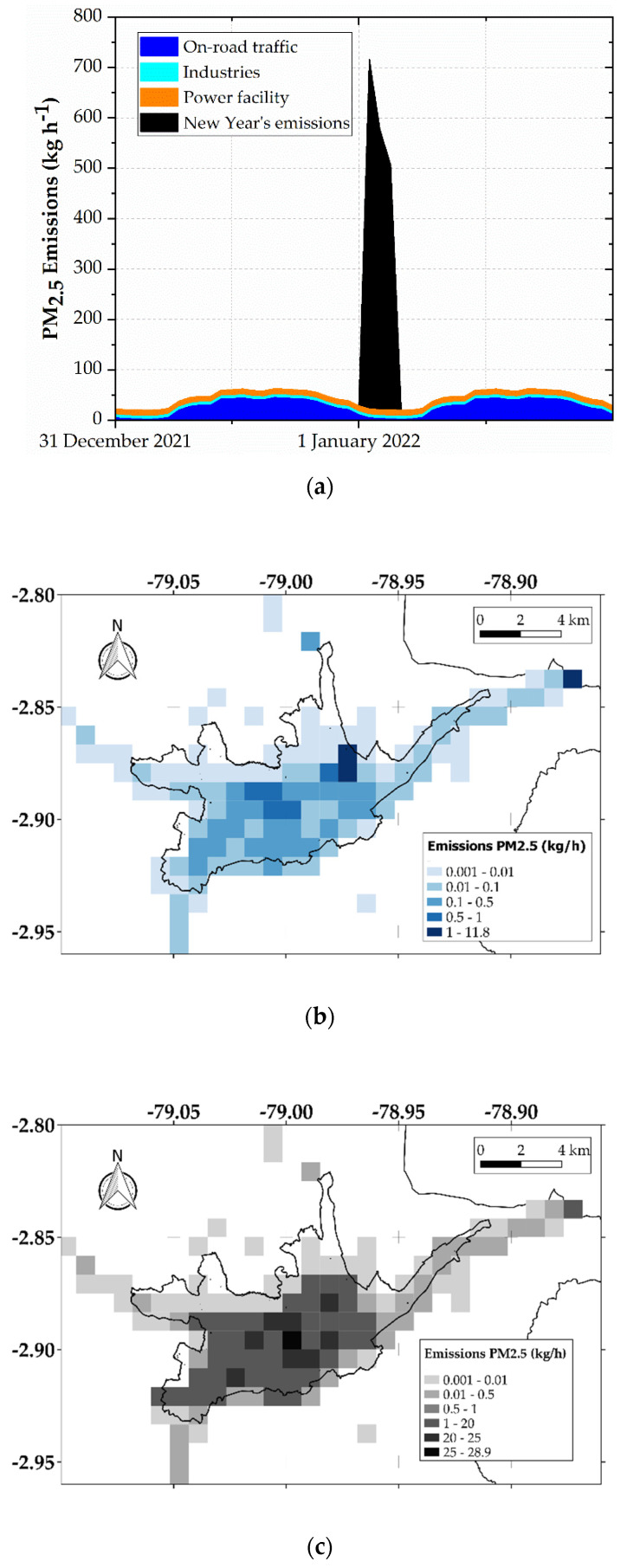
(**a**) Hourly emissions from 31 December 2020 to 1 January 2022 from background sources (on-road traffic, industries, and power facility) and New Year’s burning activities. (**b**) Gridded emissions on 1 January 2022 at 00:00 LT from background sources. (**c**) Gridded emissions on 1 January 2022 at 00:00 LT from background sources and New Year’s burning activities.

The total estimated emission due to the burning of fireworks and puppets was 1.73 t PM_2.5_, corresponding to an average emission factor of 2.7 g PM_2.5_ per inhabitant. We distributed the total emission over 1 January 2022 at 00:00 LT, 1 January 2022 at 01:00 LT, and 1 January 2022 at 02:00 LT, assuming percentages of 40%, 32%, and 28%, respectively. Then, the hourly emissions were spatially distributed in the modeling grid (Section 2.3), based on the population density map and index of unsatisfied needs [33]. The black component of Figure 2a indicates the total PM_2.5_ hourly emissions associated with the use of fireworks and puppets burning, starting on 1 January at 00:00 LT. Figure 2b indicates the PM_2.5_ gridded emissions on 1 January 2022 at 00:00 LT. Figure 2a also indicates the hourly emissions from on-road traffic, industries, and the power facility, as background PM_2.5_ sources for modeling. Figure 2b,c depict the gridded emissions on 1 January 2022 at 00:00 LT from background sources and from New Year’s burning activities, respectively.

### 2.3. Approach for Modeling the Dispersion of PM_2.5_

We used the 3D Eulerian Weather Research and Forecasting with Chemistry (WRF-Chem 3.2) [43] to model meteorology and PM_2.5_ dispersion in Cuenca on 31 December 2021 and 1 January 2022. WRF-Chem is a last-generation non-hydrostatic model used for numerical modeling and solving equations of atmospheric motion from global to local scales. Meteorological simulations were performed through a master domain of 70 × 70 cells (27 km each) and 3 nested subdomains. The cells of the third domain (100 × 82) have 1 km of resolution and cover the region of Cuenca (Figure 1c). For the third subdomain, hourly PM_2.5_ emissions were applied to WRF-Chem. As background sources, we used the PM_2.5_ emissions from on-road traffic, industries, and the power facility in the northeast of the city corresponding to a festival day, the hourly emissions of which are indicated in Figure 2a. The PM_2.5_ emissions on 1 January 2022 totaled 2.8 t d^−1^, corresponding with the 1.7 t d^−1^ (Section 2.2), 0.6 t d^−1^, 0.3 t d^−1^, and 0.1 t d^−1^ of the New Year’s combustion activities, on-road traffic, power facility, and industries, respectively. Background emissions were obtained from the last emission inventory from Cuenca. Initial and boundary conditions were generated from the final NCEP FNL operational global analysis data [44]. Table 1 summarizes the options selected for modeling.

First, we assessed the performance for modeling the hourly temperature and wind speeds at the surface through the following metrics:

(2) $$GE=\frac{1}{N}\sum_{i=1}^{N}\left|Pi-Oi\right|,$$

(3) BIAS=Pm−Om,

(4) $$RMSE=\sqrt{\frac{1}{N}\sum_{i=1}^{N}{\left(Pi-Oi\right)}^{2}},$$

*GE*: gross error; *RMSE*: root-mean-square error; *N*: number of values; *Pm*: mean modeled value; *Om*: mean observed value; *Pi*: modeled value; *Oi*: observed value. Table 2 indicates the benchmark values for these indicators.

According to the WHO guidelines, we obtained the maximum mean PM_2.5_ concentrations during 24 consecutive hours from 31 December 2021 to 1 January 2022, using the records provided by each of the six stations currently measuring PM_2.5_ (Figure 1d). Then, the modeling values at the location of each station were compared with the corresponding records. We considered that the model captured the observed records if the difference was less than 50%.

After we assessed the influence of advancing the time of burning activities, assuming these emissions began on 31 December 2021 at 21:00 LT and on 31 December 2021 at 18:00 LT. The results of these two scenarios were compared with the modeled values of the reference scenario, corresponding to the New Year’s emissions beginning on 1 January 2022 at 00:00 LT. In addition, using the emissions estimated for 31 December 2021 and 1 January 2022, we conducted similar simulations for the previous five years (2016–2017, 2017–2018, 2018–2019, 2019–2020, and 2020–2021).

## 3. Results and Discussion

### 3.1. PM_1_ and PM_2.5_ Records on 31 December 2021 and 1 January 2022

**Figure 3:**
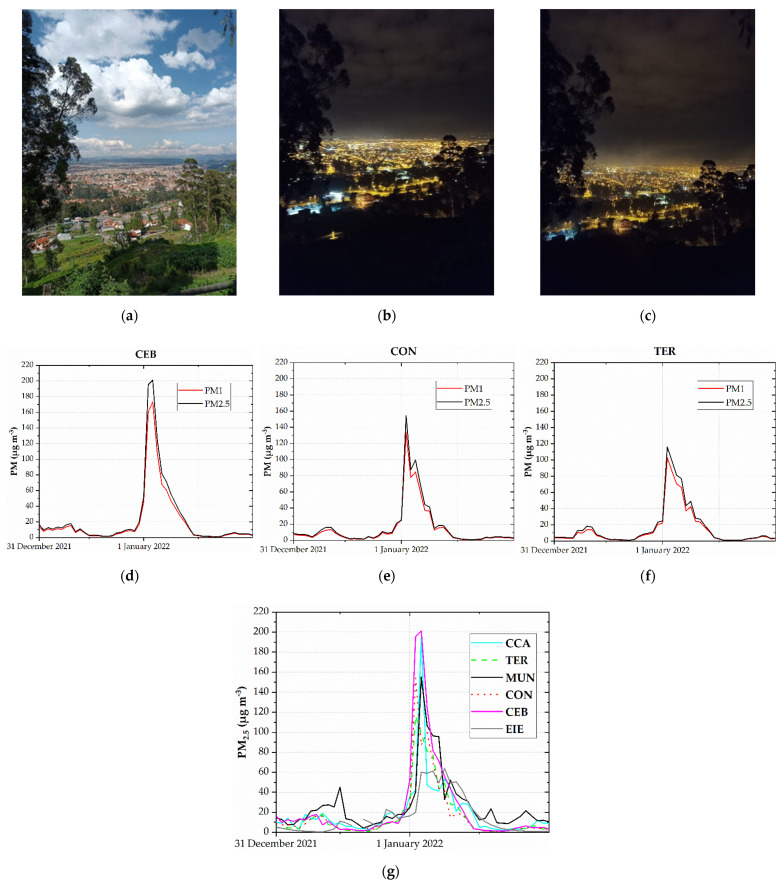
View of the city of Cuenca: (**a**) during the afternoon of 31 December 2021; (**b**) approximately 5 min before the midnight of 31 December 2021; (**c**) after the first 30 min of 1 January 2022. Hourly PM_1_ and PM_2.5_ concentrations on 31 December 2021 and 1 January 2022 measured at the CEB (**d**), CON (**e**), and TER (**f**) stations. (**g**) Hourly PM_2.5_ concentrations on 31 December 2021 and 1 January 2022.

Figure 3 depicts the views of Cuenca during the afternoon of 31 December 2021, at approximately midnight, and after the first 30 min of 1 January 2022. In addition, Figure 3 shows the hourly PM_1_ and PM_2.5_ concentrations. The maximum PM_2.5_ hourly concentrations ranged between 63.7 and 201 µg m^−3^. The highest PM_2.5_ level (201.0 µg m^−3^) was measured by the CEB station on 1 January 2022 at 01:00 LT. On average, on 1 January 2022, the ratio between PM_1_ and PM_2.5_ at the CEB, CON, and TER stations ranged from 0.84 to 0.88. This ratio is similar to the value (0.89) obtained from the PM_1_ and PM_2.5_ records reported by Majumdar et al. (2017) in relation to a firecracker bursting episode during Diwali (India) [52]. The maximum 24 h mean PM_2.5_ concentrations measured in the six stations ranged between 27.3 and 40.6 µg m^−3^. These concentrations were higher than 15.0 µg m^−3^, the current recommended value by the WHO [53]. From 2016 to 2021, the MUN station measured 8.1 to 37.8 µg m^−3^. For comparison purposes, from 2016 to 2020, the maximum 24 h mean PM_2.5_ concentrations on 1 January measured in Quito, the capital of Ecuador, ranged between 13.4 and 121.5 µg m^−3^ [54], indicating that air pollution due to New Year’s emissions was of a higher magnitude there than in Cuenca.

### 3.2. Modeling PM_2.5_ Dispersion on 31 December and 1 January

#### 3.2.1. Period from 31 December 2021 to 1 January 2022

**Figure 4:**
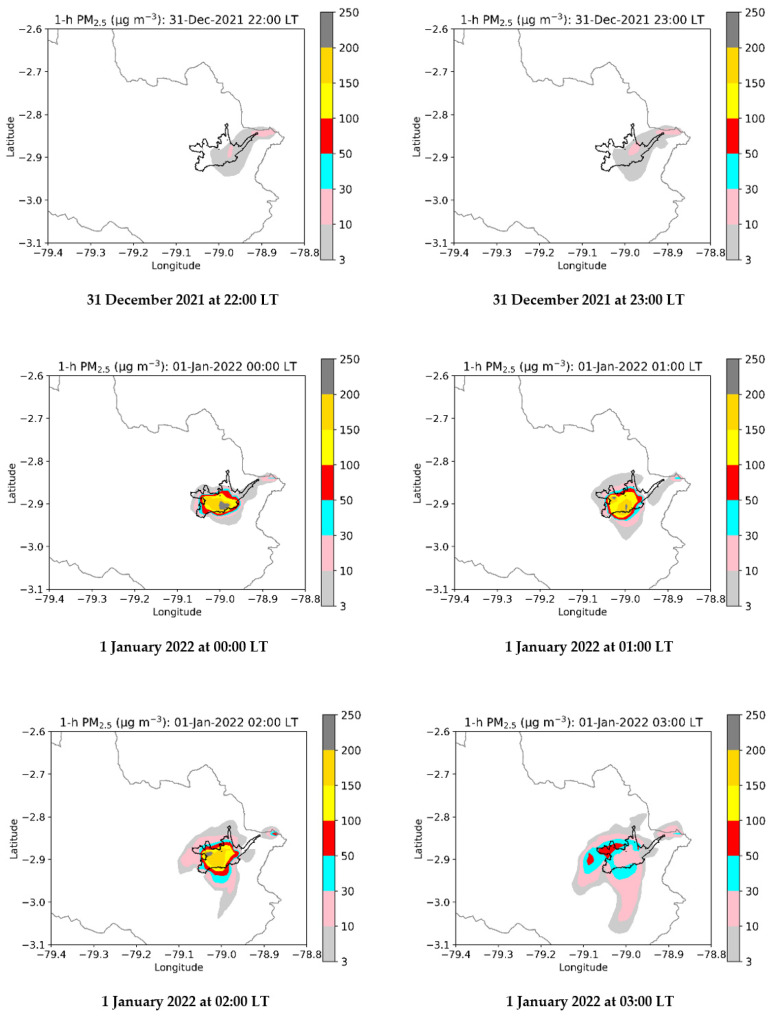
Modeled maps of hourly PM_2.5_ concentrations from 31 December 2021 at 22:00 LT to 1 January 2022 at 03:00 LT. Reference scenario: New Year’s emissions beginning on 1 January 2022 at 00:00 LT.

**Figure 5:**
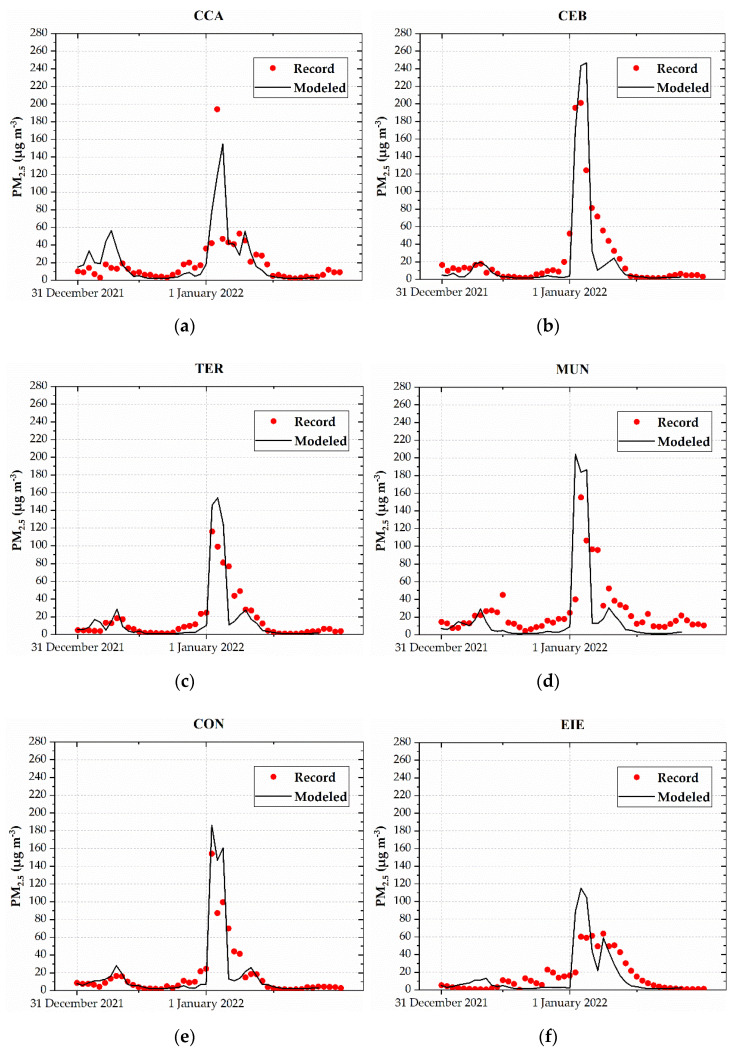
Hourly PM_2.5_ records and modeled concentrations from 31 December 2021 to 1 January 2022: the (**a**) CCA, (**b**) CEB, (**c**) TER, (**d**) MUN, (**e**) CON, and (**f**) EIE stations.

**Figure 6:**
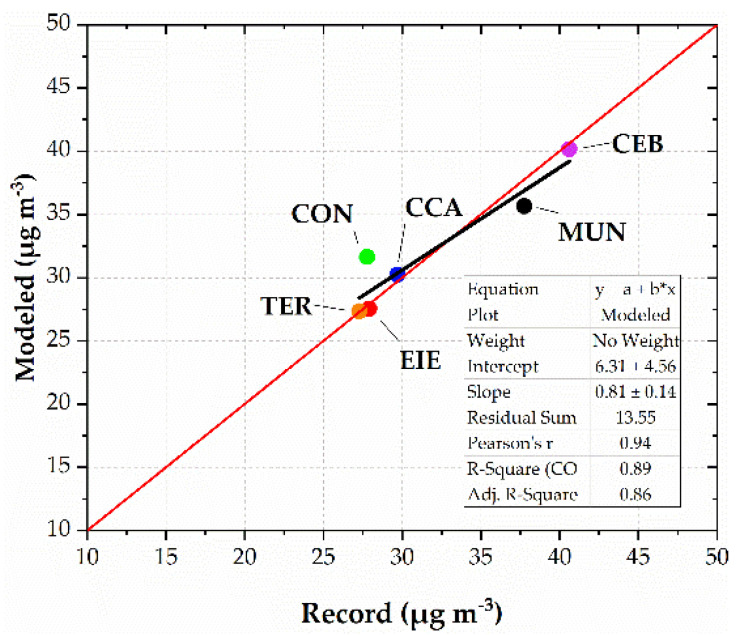
Maximum 24 h mean PM_2.5_ records and modeled concentrations from 31 December 2021 to 1 January 2022. Dots and nomenclature correspond to the six stations measuring PM_2.5_ concentrations. The black line indicates the linear fit between records and modeled values. The red line corresponds to a perfect fit.

**Figure A1:**
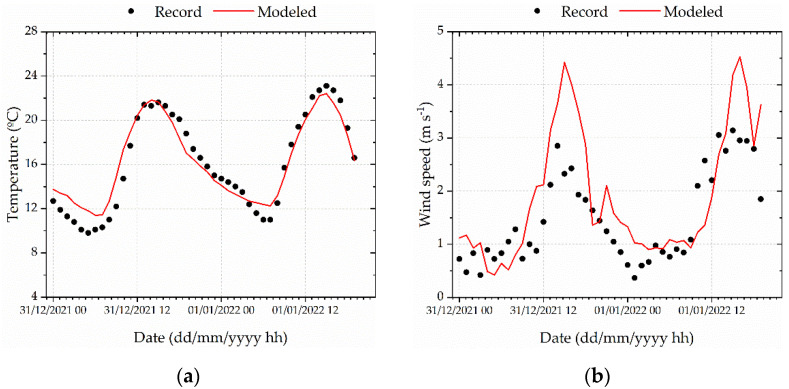
Hourly meteorological records versus modeled values on 31 December 2021 and 1 January 2022 (MUN station): (**a**) temperature and (**b**) wind speed.

Appendix A and Figure A1 indicate the assessment of modeled temperature and wind speed. Figure 4 shows the model maps of PM_2.5_ from 31 December 2021 at 23:00 LT to 1 January 2022 at 03:00 LT. The influence of emissions at 00:00 LT, with modeled concentrations up to 250 µg m^−3^ in the urban area, is clear. Modeled levels began to decrease on 1 January 2022 at 03:00 LT, while PM_2.5_ was dispersed toward the south and southwest at concentrations of up to 50 µg m^−3^. Figure 5 compares the hourly PM_2.5_ records with the corresponding modeling levels at the six stations. In general, the modeled values captured the trend of records. Figure 6 compares the PM_2.5_ (maximum 24 h mean) records and the corresponding computed values at each station. Differences between records and modeled values ranged between 0.3% and 13.9%. The linear fit reached a value of 0.89 for the coefficient of determination, highlighting the strength of the relationship between records and modeled values [54]. These metrics indicated that the simulation of PM_2.5_ dispersion was properly performed.

Although it was a component with a high level of uncertainty, the results indicated that the total PM_2.5_ emissions were acceptable, both in magnitude and in their temporal and spatial distribution. Therefore, it is feasible that these emissions were used for the numerical experiment to simulate the effects of PM_2.5_ levels, in case the emissions started before midnight on 31 December 2021, and to estimate the changes in PM_2.5_ concentrations for the meteorological conditions corresponding to 31 December and 1 January of the previous years.

#### 3.2.2. Modeling the Effect of Advancing the Time of Burning Activities

**Figure A2:**
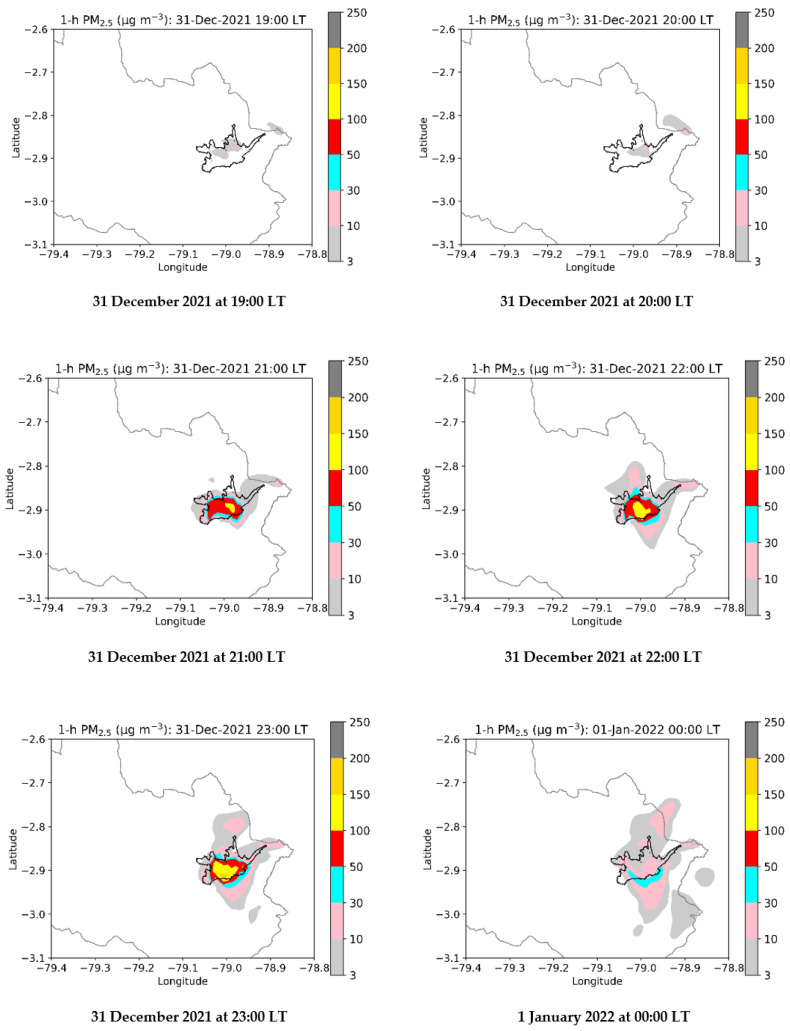
Modeled maps of hourly PM_2.5_ concentrations from 31 December 2021 at 19:00 LT to 1 January 2022 at 00:00 LT. Scenario assuming that New Year’s emissions began on 31 December 20221 at 21:00 LT.

**Figure A3:**
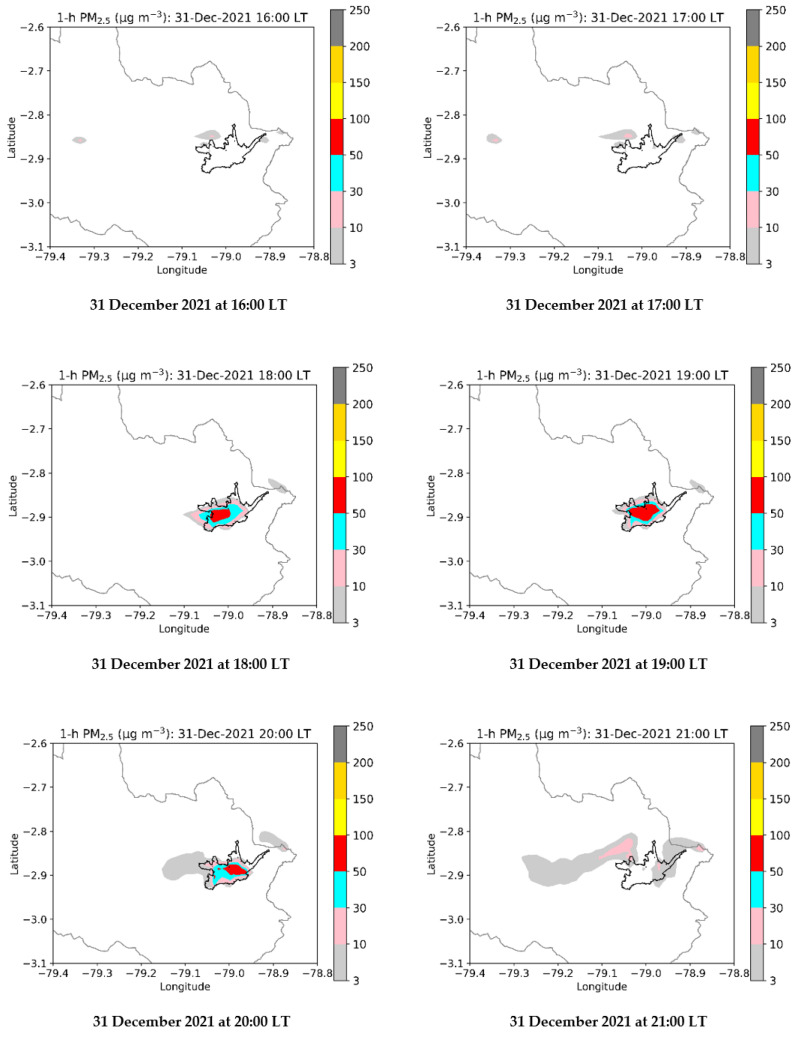
Modeled maps of hourly PM_2.5_ concentrations from 31 December 2021 at 16:00 LT to 31 December 2021 at 21:00 LT. Scenario assuming that New Year’s emissions began on 31 December 20221 at 18:00 LT.

Figure A2 and Figure A3 (Appendix B) depict the modeled maps of hourly PM_2.5_ concentrations on 31 December 2021 to 1 January 2022, assuming that emissions began on 31 December 2021 at 21:00 LT and 31 December 2021 at 18:00. For the scenario with emissions at 21:00, the maximum modeled values at 21:00, 22:00, and 23:00 LT were up to 150 µg m^−3^. For the scenario with emissions at 18:00, maximum concentrations were up to 100 µg m^−3^.

**Figure 7:**
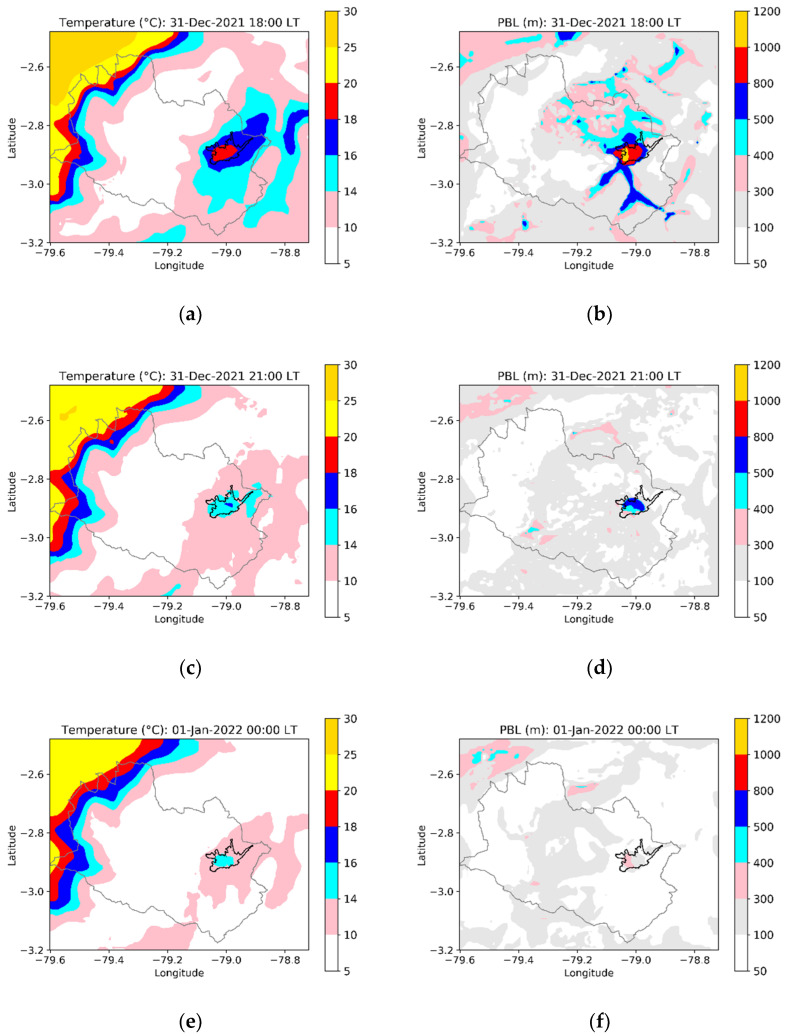
Modeled maps of temperature and planetary boundary layer (PBL) depth for selected hours: 31 December 2021 at 18:00 LT (**a**,**b**), 31 December 2021 21:00 LT (**c**,**d**), and 1 January 2022 at 00:00 LT (**e**,**f**).

**Figure 8:**
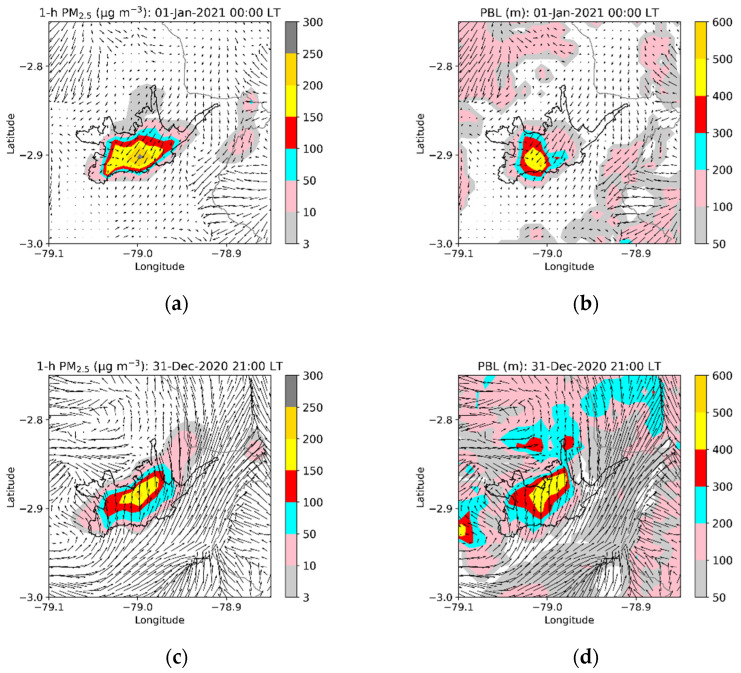
Modeled maps of PM_2.5_ and planetary boundary layer (PBL) depth. Reference scenario: New Year’s emissions starting on 1 January 2021 at 00:00 LT (**a**,**b**). Scenario with New Year’s emissions starting on 31 December 2020 at 21:00 LT (**c**,**d**).

Figure 7 indicates the modeled temperature and PBL maps for 31 December at 18:00 LT, 31 December at 21:00 LT, and 1 January 2022 at 00:00 LT. At 18:00, the temperature in the urban area varied between 15 and 20 °C (Figure 7a). At 21:00, values between 13 and 15 °C were computed around the historic center (Figure 8c). At 00:00, the temperature ranged between 13 and 15 °C in almost all of the urban area (Figure 7e). The decrease in temperature accompanied decreases in PBL. At 18:00, the PBL over the urban area ranged between 800 and 1200 m (Figure 7b); and by 21:00, it decreased to 800 m. At 00:00, the PBL dropped to 300 and 400 m. The higher PBL depths at 18:00 and 21:00 on 31 December imply larger volumes of the atmosphere to disperse the PM_2.5_ emissions, compared with the condition at 00:00, therefore explaining why lower concentrations were computed with emissions beginning at 18:00 and 21:00.

Table 3 shows the modeled maximum 24 h PM_2.5_ levels for the reference scenario (emissions on 1 January at 00:00 LT) and for the scenario with emissions on 31 December at 21:00 LT. Results cover the last six years. Mean values for the reference scenario at the six stations ranged between 32.1 and 70.9 µg m^−3^. For emissions on 31 December at 21:00 LT, values ranged between 19.6 and 37.9 µg m^−3^. Mean reductions ranged between 2.3% (2.6 µg m^−3^) and 40.7% (33.0 µg m^−3^). This range of reductions is directly related to different atmospheric conditions to disperse the emissions of PM_2.5_.

Table 4 shows the modeled maximum PM_2.5_ levels for the reference scenario and for the alternative with emissions on 31 December at 18:00 LT. For this alternative, mean values ranged between 15.3 and 31.9 µg m^−3^. Mean reductions compared with the reference scenario ranged between 21.4% (8.5 µg m^−3^) and 61.0% (50.0 µg m^−3^). The results of the simulations for 2016 to 2021 indicated clear benefits for the scenario with emissions starting at 18:00. The benefits were lower, or even minimal (2020–2021), for the scenario that considers emissions beginning at 21:00.

Applying the decrease of 0.65% in mortality per 10 µg m^−3^ decrease in PM_2.5_ concentrations [53,55], the emissions on 31 December at 21:00 implied reductions between 0.17% and 2.15% in all non-accidental mortality. The emissions on 31 December at 18:00 resulted in decreases between 0.55% and 3.25%.

The results of 31 December 2020 (2020–2021) with emissions at 21:00 show an average decrease of 2.6 µg m^−3^. At the EIE station, the model indicated an average decrease of 14.0 µg m^−3^, although an increase of 11.6 µg m^−3^ was obtained at the CCA station (Table 3). These results deserve specific analysis. Figure 8 presents the modeling results of PM_2.5_ concentrations and the corresponding PBL heights on 1 January 2021 at 00:00 LT (reference scenario) and on 31 December 2020 at 21:00 LT (scenario of New Year’s emissions starting at 21:00). The figure details the urban area results and includes surface wind vectors.

For the reference scenario, on 1 January 2021 at 00:00 LT, wind was mainly generated from the northeast, transporting the pollutants to the southwest. In addition, in most of the urban area, PBL heights between 100 and 500 m were modeled, with values greater than 200 m in the historic center, an area in which concentrations of up to 300 µg m^−3^ were obtained. In the northern part of the urban area, concentrations between 10 and 100 µg m^−3^ were modeled.

For the scenario with New Year’s emissions starting at 21:00 LT, the wind moved from the south in the urban area, transporting emissions to the north. The PBL presented values greater than 200 m in a larger area than the values on 1 January 2021 at 00:00 LT. However, concentrations of up to 200 µg m^−3^ were obtained in the northern part of the urban area. This increase was modeled in the coverage areas of the CCA and CEB stations. The numerical experiment indicates that wind transports the emissions to the north of the historic center. Although the greater heights of the PBL at the CCA and CEB stations allow for more efficient dispersion, the transport of PM_2.5_ from the historic center has a more significant effect and, consequently, higher concentrations than the reference scenario. This particular result highlights the complexity of the interaction between emissions and meteorological conditions, and indicates that wind direction can promote local increases in PM_2.5_ levels, despite the greater height of the PBL. Although the model’s performance in capturing the temperature and wind speed for 31 December 2021 and 1 January 2022 was evaluated in this study, this result highlights the need to include the evaluation of the modeled wind direction in the future. It also highlights the importance of carrying out specific evaluations for other regions or cities due to their topographic conditions, geographic location, and the temporal/spatial configuration of their emissions.

In future, the competent authority could start with the generation and delivery of the forecasted PM_2.5_ concentrations, considering that emissions will occur on 1 January at 00:00 LT. This information will act as a warning and recommendation for preventive measures to reduce exposure, especially for vulnerable populations or people with pre-existing diseases. Suppose the forecast indicates atmospheric conditions with high stability and, therefore, high PM_2.5_ concentrations. In that case, the competent authority could request that citizens avoid or reduce emissions arising from the use of fireworks, firecrackers, and burning puppets and limit the time they spend outdoors.

Based on forecasted PM_2.5_ levels, the competent authority could also inform citizens about the benefits of advancing emission activities before midnight on 31 December. In this sense, this information can promote the collaboration of at least part of the population. Implementing a forecasting system based on the approach described in this study is challenging. For this purpose, it is necessary to have trained personnel and sufficient computational resources to have the modeled results sufficiently in advance.

In future, the performance of numerical models to capture unusual conditions, such as situations of strong atmospheric stability, should be investigated. The influence of the initial conditions also deserves dedicated research, because the performance of numerical models for meteorological forecasting tends to gradually degrade after a specific time scale (time after the initial conditions) [21]. Weather forecasting at small scales, as in the case of Cuenca, deteriorates more rapidly than at larger scales. In addition, the first hours of a weather forecast are relatively useless while the numerical model adjusts to imbalances in the initial conditions.

### 3.3. Limitations of This Study and Future Activities

Our estimation of the New Year’s emissions (1.73 t PM_2.5_) represents approximately 0.2% of the total PM_2.5_ emissions (907 t per year) reported in the emission inventory of Cuenca for 2014 [34]. This value is consistent with the criteria of the Environment Agency of Austria [56], an entity which indicated that on New Year’s Eve, fireworks account for less than 0.6% of the annual PM_2.5_ emissions. However, we highlight the uncertainty of the emissions, and the limitation or lack of information in Ecuador to characterize the corresponding emission activities through a bottom-up approach. Likewise, the use of emission factors taken from the literature contribute to uncertainty, as values could differ for the altitude of Cuenca (2500 m.a.s.l.), in which the availability of oxygen in the atmosphere is 25% lower compared to sea levels [57].

We assumed that the emissions generated by the use of fireworks and the burning of puppets occurred during the first three hours of 1 January 2022. However, it was observed that the use of fireworks mainly occurs at 00:00 LT, and although the burning of puppets also mostly begins at 00:00, its emissions can last for several hours. The estimation of PM_2.5_ emissions must be improved by collecting information that allows for the better characterization of activity levels and emission factors. This is a challenging task because emissions can differ year to year, both in magnitude and even location. Particulate material composition must also be characterized in terms of heavy metals, black, and elemental carbon, and monitoring the levels of particulate matter in the number of particles per unit volume. In addition, it is necessary to study the influence of relevant PM_2.5_ sources, as industries with permanent and significant emissions are simultaneous with emissions associated with the arrival of a new year.

The Andean Ecuadorian region has poor coverage of radiosonde observations. This limitation implies an incomplete description of the atmospheric conditions for meteorological and air quality parameters. Although in Cuenca atmospheric monitoring has improved, the air quality network was predominantly designed to satisfy national regulation requirements, covering only surface measurements. Vertical observations need to be promoted to improve our understanding of atmospheric conditions and their interaction with air pollutants. This information will allow for more complete studies about modeling performance and improved results for air quality management.

We explored a novel and promising approach for controlling the effects of PM_2.5_ emissions due to New Year’s burning activities, in an Andean city with complex topography. Other ways of reducing PM_2.5_ emissions, such as limiting or controlling the sale of fireworks and the burning of puppets, should be assessed. These options have been analyzed in other regions (e.g., [31,58]). However, we did not find studies based on the approach used in this study.

Animated .gif files of the modeled PM_2.5_ dispersion for emissions on 1 January at 00:00 LT (Figure S1), and for the alternatives on 31 December 2021 at 21:00 LT (Figure S2) and 31 December 2021 at 18:00 LT (Figure S3), are available in the Supplementary Materials. These files were created using the integrated data viewer tool [59].

## 4. Conclusions and Summary

We modeled the dispersion of PM_2.5_ emitted into the atmosphere, due to the use of fireworks and other combustion activities associated with the arrival of a new year in an Andean Ecuadorian city. Our numerical experiments indicated that one way to reduce the effects on air quality is advancing the time of emissions to hours before midnight on 31 December. Our modeling approach applied to the last six years and provided mean decreases between 2.3% and 40.7% in the 24 h mean PM_2.5_ concentrations, if the emissions begin on 31 December at 21:00 LT. The decrease ranged between 21.4% and 61.0% if emissions began on 31 December at 18:00 LT. The reduction in concentrations is produced by better atmospheric conditions to disperse PM_2.5_. Higher benefits can be obtained if the emissions take place at times of higher planetary boundary layer depths, when atmospheric conditions promote the pollutant’s dispersion. This approach can be applied to assess the benefits of advancing New Year’s emissions in other regions, based on their topographic conditions, geographic location, and the temporal/spatial configuration of their emissions.

In the future, the performance of numerical models to capture unusual conditions, such as situations of strong atmospheric stability, should be investigated. Moreover, the influence of the initial conditions deserves a dedicated study. The particulate material composition must be characterized in terms of heavy metals, black and elemental carbon contents. Monitoring should cover the levels of particulate matter in the number of particles per unit volume. In addition, it is necessary to have a dedicated study investigating the influence of relevant PM_2.5_ sources in industries with permanent and significant emissions, with the emissions associated with the arrival of a new year. Vertical observations need to be promoted in the Andean Ecuadorian region, to improve understanding of atmospheric conditions and their interaction with air pollutants. The estimation of New Year’s PM_2.5_ emissions must be improved by collecting information that allows for better characterization of the activity levels and emission factors.

## Figures and Tables

**Table 1:** Options for modeling the dispersion of PM_2.5_ in Cuenca (WRF-Chem 3.2).

Component	Option	Scheme/Model and Reference
Microphysics (mp_physics)	2	Lin et al. [45]
Longwave Radiation (ra_lw_physics)	1	RRTM [46]
Shortwave Radiation (ra_sw_physics)	2	Goddard [47]
Surface Layer (sf_clay_physics)	1	MM5 similarity [48]
Land Surface (sf_surface_physics)	2	Noah Land Surface Model [49]
Planetary boundary layer (bl_pbl_physics)	1	Yonsei University [50]
Cumulus parameterization (cu_physics)	0	Without parameterization

**Table 2:** Metrics for modeling temperature and wind speed (WRF-Chem 3.2) [51].

Parameter	Indicator	Benchmark
Hourly surface temperature	GE	<2 °C
	BIAS	<±0.5 °C
Hourly wind speed at 10 m above the surface	RMSE	<2 m s^−1^
	BIAS	<±0.5 m s^−1^

**Table 3:** Effect of advancing the time of burning puppets and fireworks. Scenario 1 January at 00:00 LT vs. 31 December at 21:00 LT.

Station	2016–2017	2017–2018	2018–2019	2019–2020	2020–2021	2021–2022
(A) Scenario 1 January at 00:00 LT: Modeled maximum PM_2.5_ levels (µg m^−3^) during 24 h						
MUN	119.6	47.8	48.1	42.7	45.9	35.6
EIE	38.2	35.5	25.0	12.2	33.2	27.5
CCA	58.4	43.2	74.0	46.6	26.4	30.2
CON	76.4	45.1	35.0	37.3	43.5	31.6
CEB	21.6	34.9	32.7	23.8	23.6	40.2
TER	111.0	36.2	33.1	33.7	34.8	27.3
Mean	70.9	40.5	41.3	32.7	34.6	32.1
(B) Scenario 31 December at 21:00 LT: Modeled maximum PM_2.5_ concentrations (µg m^−3^) during 24 h						
MUN	55.8	37.3	37.4	23.9	38.7	22.4
EIE	27.4	20.6	25.6	11.5	19.2	14.8
CCA	44.5	44.0	42.6	35.5	38.1	21.7
CON	39.5	31.5	34.9	18.9	36.3	20.9
CEB	14.8	35.3	33.6	19.6	32.8	19.0
TER	45.3	32.9	23.3	20.2	27.0	18.8
Mean	37.9	33.6	32.9	21.6	32.0	19.6
Difference: (B) − (A), (µg m^−3^)						
MUN	−63.8	−10.5	−10.7	−18.8	−7.2	−13.2
EIE	−10.7	−14.9	0.6	−0.7	−14.0	−12.7
CCA	−13.8	0.8	−31.4	−11.1	11.6	−8.6
CON	−36.9	−13.6	−0.1	−18.4	−7.1	−10.7
CEB	−6.8	0.4	0.8	−4.2	9.2	−21.1
TER	−65.7	−3.4	−9.8	−13.5	−7.8	−8.5
Mean	−33.0	−6.9	−8.5	−11.1	−2.6	−12.5
Percentage of difference: ((B) − (A)/(A)) × 100, (%)						
MUN	−53.3	−21.9	−22.3	−44.1	−15.7	−37.1
EIE	−28.1	−42.0	2.2	−5.8	−42.1	−46.3
CCA	−23.7	1.9	−42.5	−23.8	44.0	−28.3
CON	−48.3	−30.2	−0.3	−49.4	−16.4	−33.8
CEB	−31.4	1.1	2.6	−17.7	38.8	−52.6
TER	−59.2	−9.4	−29.7	−40.1	−22.5	−31.2
Mean	−40.7	−16.7	−15.0	−30.1	−2.3	−33.2

**Table 4:** Effect of advancing the time of burning puppets and fireworks. Scenario 1 January at 00:00 LT vs. 31 December at 18:00 LT.

Station	2016–2017	2017–2018	2018–2019	2019–2020	2020–2021	2021–2022
(A) Scenario 1 January at 00:00 LT: Modeled maximum PM_2.5_ levels (µg m^−3^) during 24 h						
MUN	119.6	47.8	48.1	42.7	45.9	35.6
EIE	38.2	35.5	25.0	12.2	33.2	27.5
CCA	58.4	43.2	74.0	46.6	26.4	30.2
CON	76.4	45.1	35.0	37.3	43.5	31.6
CEB	21.6	34.9	32.7	23.8	23.6	40.2
TER	111.0	36.2	33.1	33.7	34.8	27.3
Mean	70.9	40.5	41.3	32.7	34.6	32.1
(B) Scenario 31 December at 18:00 LT: Modeled maximum PM_2.5_ concentrations (µg m^−3^) during 24 h						
MUN	22.8	36.6	20.8	21.2	22.5	15.7
EIE	15.0	18.2	12.7	10.2	11.7	15.7
CCA	33.1	45.1	33.3	27.5	27.9	19.6
CON	21.6	30.6	19.8	20.3	20.2	14.5
CEB	16.5	27.0	14.6	22.2	17.0	13.4
TER	16.0	34.2	13.6	15.0	19.3	12.7
Mean	20.8	31.9	19.1	19.4	19.8	15.3
Difference: (B) − (A), (µg m^−3^)						
MUN	−96.8	−11.3	−27.3	−21.5	−23.4	−19.9
EIE	−23.1	−17.3	−12.3	−2.1	−21.4	−11.8
CCA	−25.3	1.9	−40.7	−19.1	1.5	−10.7
CON	−54.9	−14.5	−15.2	−17.0	−23.2	−17.1
CEB	−5.1	−7.9	−18.1	−1.6	−6.6	−26.7
TER	−95.0	−2.1	−19.5	−18.7	−15.5	−14.6
Mean	−50.0	−8.5	−22.2	−13.3	−14.8	−16.8
Percentage of difference: ((B) − (A)/(A)) × 100, (%)						
MUN	−80.9	−23.6	−56.8	−50.3	−50.9	−55.9
EIE	−60.7	−48.7	−49.2	−17.0	−64.7	−42.8
CCA	−43.3	4.4	−55.0	−41.0	5.6	−35.4
CON	−71.8	−32.1	−43.5	−45.5	−53.4	−54.1
CEB	−23.5	−22.6	−55.3	−6.8	−28.0	−66.6
TER	−85.5	−5.7	−59.0	−55.4	−44.5	−53.5
Mean	−61.0	−21.4	−53.1	−36.0	−39.2	−51.4

## Data Availability

Not applicable.

## Supplementary Materials

The following supporting information can be downloaded at: https://www.mdpi.com/article/10.3390/toxics10050264/s1, Figure S1: Modeled PM_2.5_ dispersion on 31 December 2021–1 January 2022. Emission beginning on 1 January 2022 at 00:00 LT. Figure S2: Modeled PM_2.5_ dispersion on 31 December 2021–1 January 2022. Emission beginning on 31 December 2021 at 21:00 LT. Figure S3: Modeled PM_2.5_ dispersion on 31 December 2021–1 January 2022. Emission beginning on 31 December 2021 at 18:00 LT.

## Appendix A

Figure A1 compares the observed and modeled temperatures at the MUN station. The modeled values captured the trend of records, which indicate higher levels (approximately 22 °C) two hours after midday, and the minimum (11 °C) on 1 January 2022 at 06:00 LT. The values of the GE and BIAS metrics were 1.0 and 0.18 °C, respectively; both values were in the corresponding benchmark ranges (Table 2). Figure 4 compares the hourly records and modeled values of wind speed. The highest records (3 m s^−1^) were measured at midday. The minimum record (0.4 m s^−1^) was measured on 1 January 2022 at 01:00 LT. The RMSE and BIAS metrics were 0.8 and 0.4 m s^−1^, respectively, which are in the corresponding benchmark ranges. Therefore, these meteorological parameters were captured by modeling, implying the dynamic of PBL depths and atmospheric stability was also acceptably modeled.
