# Supplementary material for: Simulating PM2.5 Concentrations during New Year in Cuenca, Ecuador: Effects of Advancing the Time of Burning Activities
Source: Toxics. 2022 May 19;10(5):264. doi: 10.3390/toxics10050264 (PMC9144387; doi:10.3390/toxics10050264)
Supplement: Supplementary file 1 [file toxics-10-00264-s001.zip › toxics-1679132-supplementary.pdf]

toxics

Supplementary Materials: Simulating PM2.5 Concentrations during New Year in Cuenca, Ecuador: Effects of Advancing the Time of Burning Activities

René Parra, Claudia Saud and Claudia Espinoza

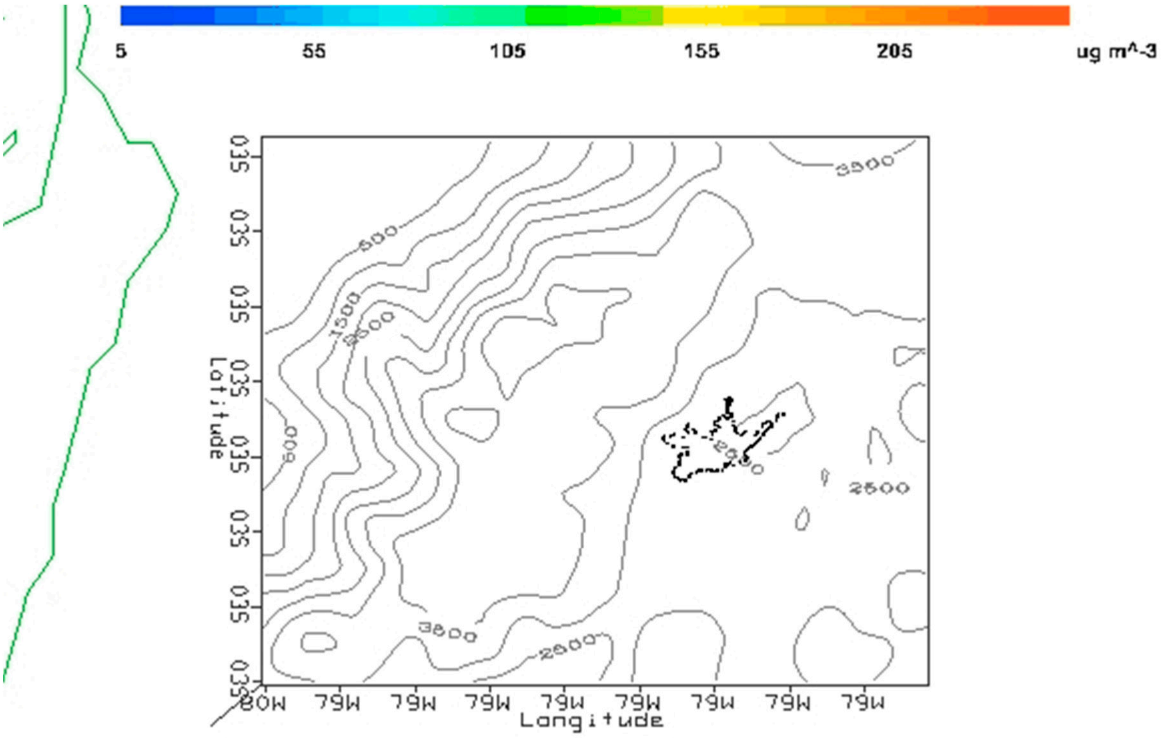

Figure S1. Modeled PM2.5 dispersion on 31 December 2021–1 January 2022. Emission beginning at 1 January 2022 00:00 LT.

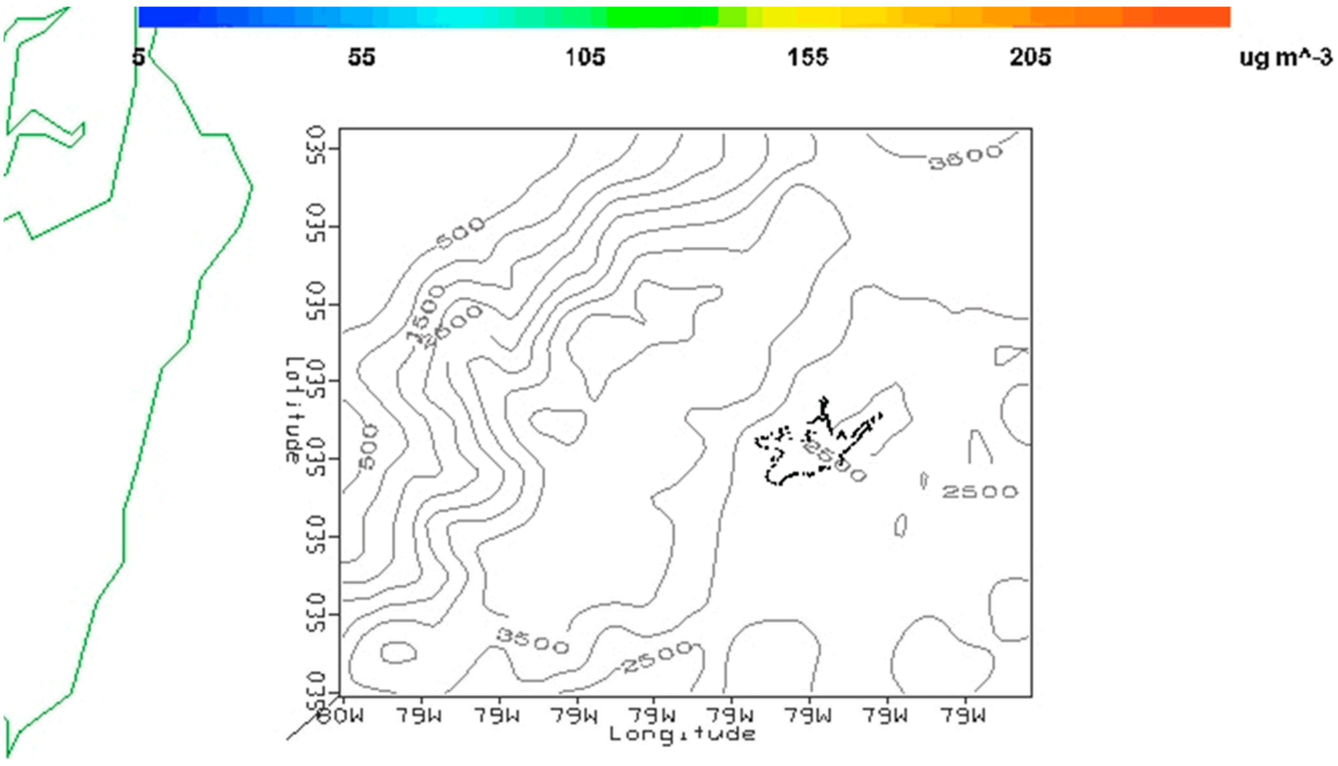

Figure S2. Modeled $PM_{2.5}$ dispersion on 31 December 2021–1 January 2022. Emission beginning at 31 December 2021 21:00 LT.

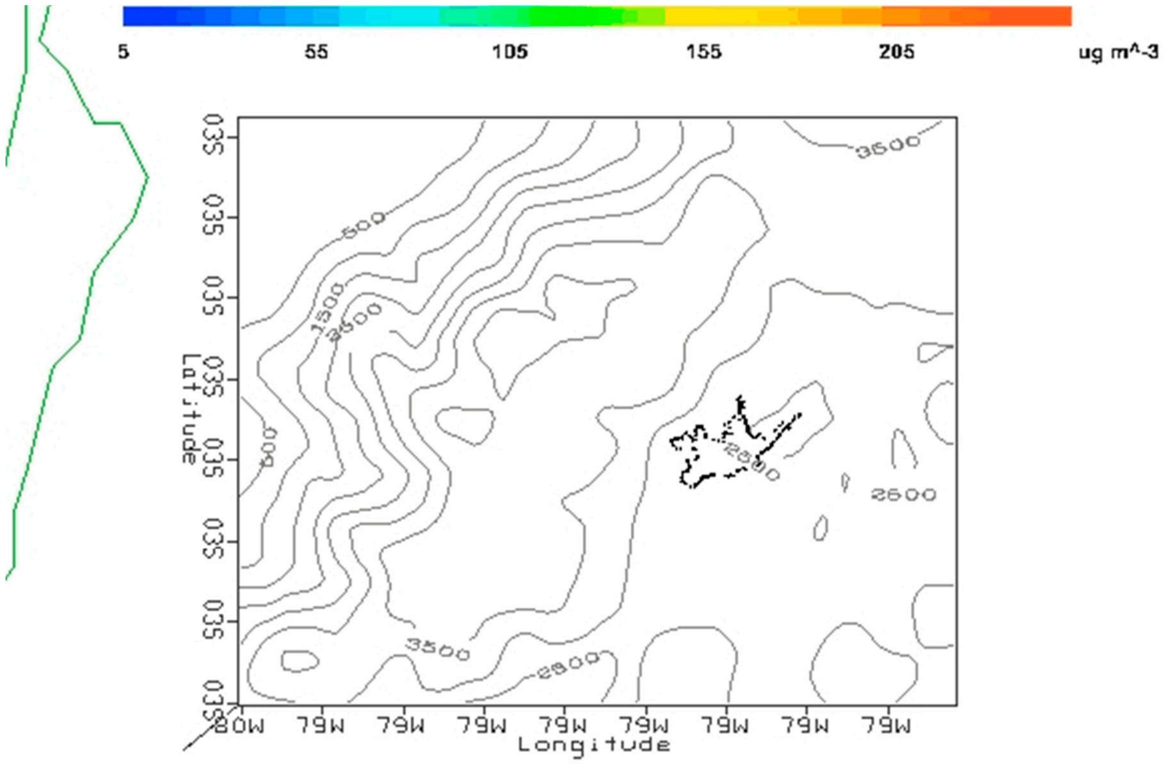

Figure S3. Modeled $PM_{2.5}$ dispersion on 31 December 2021–1 January 2022. Emission beginning at 31 December 2021 18:00 LT.
